# Retrotransposition in tumors and brains

**DOI:** 10.1186/1759-8753-5-11

**Published:** 2014-04-07

**Authors:** John L Goodier

**Affiliations:** 1 McKusick-Nathans Institute for Genetic Medicine, Johns Hopkins University School of Medicine, 733 N. Broadway, Baltimore, MD 21205, USA

**Keywords:** Alu, Cancer, Mosaicism, Mutation, Neuron, Retrotransposon, Somatic, SVA, Tumor

## Abstract

LINE-1s (L1s), the only currently active autonomous mobile DNA in humans, occupy at least 17% of human DNA. Throughout evolution, the L1 has also been responsible for genomic insertion of thousands of processed pseudogenes and over one million nonautonomous retrotransposons called SINEs (mainly Alus and SVAs). The 6-kb human L1 has a 5′- untranslated region (UTR) that functions as an internal promoter, two open reading frames—ORF1, which encodes an RNA-binding protein, and ORF2, which expresses endonuclease and reverse transcriptase activities—and a 3′-UTR which ends in a poly(A) signal and tail. Most L1s are molecular fossils: truncated, rearranged or mutated. However, 80 to 100 remain potentially active in any human individual, and to date 101 *de novo* disease-causing germline retrotransposon insertions have been characterized. It is now clear that significant levels of retrotransposition occur not only in the human germline but also in some somatic cell types. Recent publications and new investigations under way suggest that this may especially be the case for cancers and neuronal cells. This commentary offers a few points to consider to aid in avoiding misinterpretation of data as these studies move forward.

## 

Retrotransposition of non-long terminal repeat (non-LTR) long interspersed nuclear elements (LINE-1 s, or L1s), as well as the mobilization *in trans* of non-autonomous short interspersed nuclear elements (SINEs) and processed pseudogenes, has built at least 50% of the human genome and remains an ongoing source of gene mutation [1,2]. As a type of “selfish DNA”, L1 activity was formerly thought to occur predominantly in germ cells, where insertions would pass to the next generation. However, in addition to the massive germline expansion of L1s that occurred during mammalian evolution, recent investigations have documented ongoing retrotransposition in selected somatic cell types, including neural progenitor cells, stem cells, early embryos, tumors and induced pluripotent stem cells [3-7]. More than 20 years ago, Miki *et al*. [8] reported the first instance of somatic retrotransposition, an L1 insertion into the adenomatous polyposis coli tumor suppressor gene of a colorectal cancer. The advent of high-throughput sequencing has made it possible to identify numerous non-germline *de novo* insertions in various kinds of cancer, as recently described in several high-profile papers [6,9-13] (Table 1).

**Table 1:** **Summary of published evidence for tumor-specific somatic retrotransposition**^**a**^

**Study**	**Cancer type**	**Tumor–normal pairs, *n***	**Tumors with somatic insertions, *n***	**Tumor-only somatic insertions, *n***				**Normal-only somatic insertions**		**Method**
				**Detected**	**Validated as tumor-specific**			**Detected**	**Validated**	
					**By PCR**	**By sequencing**				
						**3′ end only**	**3′ + 5′ ends**			
Iskow *et al*. [6]	Lung	20	6	L1: 9	8/9	8	0	0		Pyrosequencing
	Brain	10	0							
Lee *et al*. [9]	Glioblastoma	16	0	L1: 183	38/39	6	2	0		Paired-end WGS
	Ovarian	9	5	Alu: 10	1/3					
	Colorectal	5	5	ERV1: 1	1/1		1			
	Prostate	7	6							
	Multiple myeloma	7	1							
	Normal (Trio)	3	0							
Solyom *et al*. [10]	Colorectal	16	13	L1: 107	69/107	34	35	12	0	L1-Seq
Shukla *et al*. [11]	Hepatocarcinoma	19	5	L1: 17	12/17	2	10	21	1	RC-Seq
				Alu: 27	0/13					
				SVA: 1	0/1					
Ewing *et al*. [12]^b^	Acute myeloid leukemia	24	0	0						Paired-end WGS
	Breast	12	0	0						
	Colorectal adenocarcinoma	5	0	0						
	Glioblastoma	15	0	0						
	Lung	19	2	GRIP: 3	0/0					
	Ovarian	10	0	0						

^a^ERV1, Endogenous retrovirus1 (PABL_A type); L1-seq, Hemi-specific PCR coupled to Illumina sequencing [14]; RC-seq, Retrotransposon capture sequencing, involving hybridization of fragmented genomic DNA to custom retrotransposon sequence capture arrays followed by deep sequencing [15]; Trio, mother, father and child; WGS, Whole-genome sequencing. ^b^Ewing *et al*. examined only gene retrocopy insertion polymorphisms (GRIPs), which are processed gene transcripts present as retrotransposed insertions in one or more individuals but absent from the reference genome.

Cancer-associated hypomethylation and elevated transcription of L1s predicted increased retrotransposition in tumors long before new insertions were detected by next-generation sequencing [16]. It is also reasonable to assume that insertions proliferate preferentially in tumors because cancer cells divide more rapidly than their normal cells of origin. Indeed, cell cycling, though not strictly required for retrotransposition, may increase its frequency [17-19]. Interestingly, investigations to date have detected new insertion events almost exclusively in tumors of epithelial cell types, some of which proliferate and turn over quickly. Carreira *et al*. [20] speculated that increased retrotransposon insertions in epithelial tumors may relate to a greater “plasticity” of epithelial cells, which are more easily reprogrammed to yield cancer or pluripotent stem cells.

Recent high-throughput sequence studies have reported tumor-specific insertions that vary greatly in number between different tumors of the same type, ranging from 0 in most instances to 106 in a single colorectal tumor identified by Lee *et al*. [9]. The application of different methodologies clearly accounts for some of this variation. Furthermore, current sequence analysis pipelines lack sensitivity to detect rare insertion events that occur late in tumor development, thus underestimating the total number of tumor-specific insertions. On the other hand, according to the scenario of Figure 1A, claims for the tumor specificity of much *de novo* retrotransposition and its absence in matched normal cells might be illusory. This scenario assumes that retrotransposition occurs at significant rates in normal somatic cells. However, in non-tumor tissue sampled in bulk, an individual new somatic insertion may be present in only a single cell, or at most several cells, among the large total population of cells sampled and consequently exist in too small a copy number to be detected. An insertion initially present within a normal cell is more easily detected once that cell by chance clonally expands as a tumor, which, upon sampling, high-throughput sequencing and PCR validation, would falsely appear to possess a tumor-only event. Thus, although tumor-specific *de novo* retrotransposition events may be underestimated, retrotransposition in normal cells is likely grossly underestimated.

**Figure 1:**
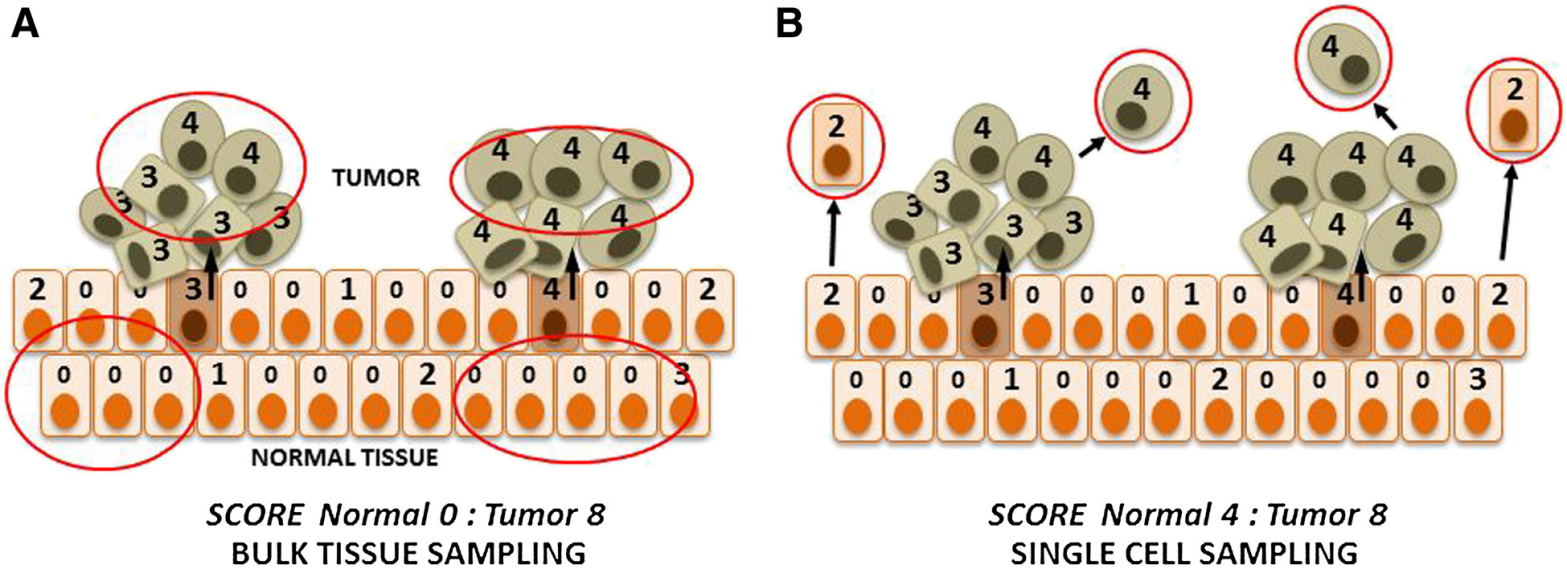
**Bulk tissue vs. single-cell detection of somatic retrotransposition. (A)** Bulk tissue sampling can underestimate the number of normal tissue retrotransposition events. **(B)** Single-cell sampling can provide truer estimates of rates of normal somatic cell retrotransposition. The numbers of unique *de novo* retrotransposon events in each cell are indicated. A minor percentage of normal epithelial cells (light brown) contain new unique insertions. Occasionally, a cancer stem cell (dark brown) gives rise to a tumor (green). Every cell of the tumor will contain the *de novo* retrotransposon insertions present in the originating stem cell. New tumor-specific events may also subsequently occur. Red circles indicate bulk **(A)** or single-cell **(B)** sampling for downstream sequencing analyses.

Solyom *et al*. [10] adopted three indirect approaches to conclude that most, if not all, of the insertions they studied occurred post-tumor initiation: (1) by finding an L1 insertion to be absent in a second section of the same tumor, (2) by detecting empty-site X chromosome alleles in males with an X chromosome tumor insertion, and (3) by querying the heterozygosity of single-nucleotide polymorphisms (SNPs) flanking an L1 insertion site (with the presence of both SNP alleles in the empty-site chromosome implying that the insertion occurred after the one-cell stage of the tumor). Because the tumor samples assayed in this study were not microdissected, however, all three pieces of evidence cited may be subject to misinterpretation because of normal tissue present within the tumor sample. Interestingly, Shukla *et al*. [11] validated by PCR a single *de novo* L1 insertion in preneoplasmic liver tissue that was absent in the corresponding hepatocellular carcinoma, although they also considered the possibility that chromosomal loss within the tumor could have deleted the insertion.

Thus, tumor-specific retrotransposon insertions occur, perhaps frequently in some tumors, but apparently vary greatly in number between different types of tumors and between individual tumors of the same cancer type. Although 80 to 100 L1s are estimated to be potentially active in any given human diploid genome [21], each particular genome can harbor its own unique, active L1s, or L1s shared between different individuals may vary in activity. My “hot” L1 may not be your hot L1; perhaps my mobilome has greater “mutational power” than yours [22,23]. Add to this fact the variability in the epigenetic state of individual active L1s, plus unknown genetic variations in the many cellular factors that associate with the L1 to affect its life cycle [24-26], and the task of assessing rates of cancer retrotransposition becomes complicated indeed.

High-throughput sequencing methods can produce false-positive results, underlining the need for validation of a significant number of the total putative somatic insertions found. As much as possible, it is important to identify 5′ as well as 3′ junctions of an insertion to confirm the presence of a poly(A) tail and a target site duplication (TSD), the hallmarks of a true retrotransposition event. A minor but significant number of L1s insert not by target-primed reverse transcription (TPRT), the standard model for L1 retrotransposition [27], but rather by an endonuclease-independent mechanism. These insertion events likely occur at preexisting DNA lesions and generate integrants lacking TSDs [28-30]. Interestingly, apparent endonuclease-independent insertions have been detected in significantly increased numbers in some tumors (8 of 35 colorectal cancer insertions reported by Solyom *et al*. [10]).

Obtaining unbiased estimates of *de novo* retrotransposition in normal as well as tumor cells is critical for understanding somatic mosaicism, cancer induction, tumor heterogeneity, and the etiology of some neurological diseases [31,32]. Evrony *et al*. [33] recently used multiple displacement amplification of single neurons isolated from the frontal cortex and caudate nucleus of three normal individuals to confirm somatic neuronal retrotransposition, but at a rate much lower (0.04 to fewer than 0.6 unique insertions per neuron) than estimates previously suggested by quantitative PCR (qPCR) analyses (a startling “theoretical” increase of about 80 L1 copies per hippocampal neuron compared with heart and liver samples [34]). Using a high-throughput method called RC-seq to analyze bulk DNA, Baillie *et al*. [15] found almost 8,000 putative somatic L1 insertions in the hippocampus and caudate nucleus of three individuals, an insertion rate much lower than Coufal *et al.*[34]^a^.

qPCR techniques are increasingly being used to conclude elevated L1 genomic copy numbers in some cell types or under some cellular conditions [34-39]. Typically, the apparent increase in retrotransposon insertions is not verified by downstream sequencing. Apart from the fact that small changes in protocol can alter the results of such sensitive qPCR analyses, an additional but untested source of bias conceivably exists: that is, the promiscuous reverse transcription (RT) of retrotransposon RNAs “free-floating” in the cell and not engaged in TPRT at a site of chromatin integration. Such ectopic RT reactions might be primed by random complementary nucleic acids or perhaps by fold-back and annealing of the L1 poly(A) tail to one of the ten homopolymeric stretches of four to seven U residues that occur across the length of the L1 RNA molecule. Fold-back self-priming of RNA has been observed for a number of RNA-dependent RNA polymerases, including reverse transcriptases [40-42]. It has also been shown that RT can initiate from internal sites within L1 RNA during endonuclease-independent insertion [28]. One would expect copy numbers of orphan L1 cDNAs generated by promiscuous RT to be higher in cells with elevated expression of L1 ORF2 or perhaps endogenous retroviral reverse transcriptases; indeed, increased RT activity in some types of neuronal and tumor cells has been reported [43-45]. Because these cDNAs are amenable to PCR amplification, qPCR-based estimates of genomic L1 insertion copy numbers in these cells using L1-specific primers would be erroneously high. This source of bias, if true, is not in disagreement with the *cis*-preference model for L1 retrotransposition. This model states that a retrotransposition-competent integration intermediate consists of L1 RNA bound *in cis* by its own encoded ORF1 and ORF2 proteins [46,47]. However, the total number of L1 protein [48] and RNA molecules present in the cell likely greatly exceed in number those bound *in cis* within *bona fide* insertion intermediates. These molecules can bind to each other *in trans* and perhaps engage in RT.

## Conclusions

The surprising discovery of frequent somatic retrotransposition has important implications for human health. To fully and accurately assess its extent, concerted efforts are needed to sequence many single cells from a large number of cell types (normal and cancerous) from many individuals, with thorough validation by PCR of individual retrotransposon inserts and capillary sequencing of the PCR products to confirm their identity (Figure 1B). Single-cell, whole-genome amplification is a recent technology and is not without biases, including allelic and locus dropouts, chimeric molecules, uneven amplification due to local variations in G-C content, and incorrect nucleotide insertions [33,49,50]. Such artifacts complicate the identification of somatic retrotransposon insertions. However, the ongoing development of new protocols, such as MALBAC (multiple annealing looping-based amplification cycles [51]), promises to reduce bias. Next-generation, single-cell sequencing protocols should help to establish the impact that ongoing L1 retrotransposition manifests in brain biology, cancer, and likely other human diseases where L1 mobilization has not yet been explored. It should also become possible to trace the history of a single somatic retrotransposon back to its origin in a specific cell type or at a specific stage of development.

## Endnote

^a^Six RC-Seq libraries, each prepared from 2.5 μg of DNA, yielded 7743 L1 insertions [15]. Since a human diploid cell contains 6.6 pg of DNA, there were on average .003 unique L1 insertions per brain cell. If most insertions were in neurons, the rate was 1 insertion per 30 to 150 neurons (the brain being variously estimated to be 10-50% neurons [52]).

## Abbreviations

ERV: Endogenous retrovirus; LINE: Long interspersed nuclear element; LTR: Long-terminal repeat; ORF: Open reading frame; RC-seq: Retrotransposon capture sequencing; SINE: Short interspersed nuclear element; SVA: SINE-R, VNTR and Alu; TSD: Target site duplication; UTR: Untranslated region; TPRT: Target-primed reverse transcription; WGS: Whole-genome sequencing.

## Competing interests

The author declares that he has no competing interests.
